# Investigation of the proton relay system operative in human cystosolic aminopeptidase P

**DOI:** 10.1371/journal.pone.0190816

**Published:** 2018-01-19

**Authors:** Hui-Chuan Chang, Camy C.-H. Kung, Tzu-Ting Chang, Shu-Chuan Jao, Yu-Ting Hsu, Wen-Shan Li

**Affiliations:** 1 Institute of Chemistry, Academia Sinica, Taipei, Taiwan; 2 Institute of Biological Chemistry, Academia Sinica, Taipei, Taiwan; 3 Doctoral Degree Program in Marine Biotechnology, National Sun Yat-Sen University, Kaohsiung, Taiwan; Weizmann Institute of Science, ISRAEL

## Abstract

Aminopeptidase P, a metalloprotease, targets Xaa-Proline peptides for cleavage [1–4]. There are two forms of human AMPP, a membrane-bound form (hmAMPP) and a soluble cytosolic form (hcAMPP)[5]. Similar to the angiotensin-I-converting enzyme, AMPP plays an important role in the catabolism of inflammatory and vasoactive peptides, known as kinins. The plasma kinin, bradykinin, was used as the substrate to conduct enzymatic activity analyses and to determine the Michaelis constant (*K*_*m*_) of 174 μM and the catalytic rate constant (*k*_*cat*_) of 10.8 s^-1^ for hcAMPP. Significant differences were observed in the activities of Y527F and R535A hcAMPP mutants, which displayed a 6-fold and 13.5-fold for decrease in turnover rate, respectively. Guanidine hydrochloride restored the activity of R535A hcAMPP, increasing the k_cat_/K_m_ 20-fold, yet it had no impact on the activities of the wild-type or Y527F mutant hcAMPPs. Activity restoration by guanidine derivatives followed the order guanidine hydrochloride >> methyl-guanidine > amino-guanidine > N-ethyl-guanidine. Overall, the results indicate the participation of R535 in the hydrogen bond network that forms a proton relay system. The quaternary structure of hcAMPP was determined by using analytical ultracentrifugation (AUC). The results show that alanine replacement of Arg535 destabilizes the hcAMPP dimer and that guanidine hydrochloride restores the native monomer-dimer equilibrium. It is proposed that Arg535 plays an important role in hcAMMP catalysis and in stabilization of the catalytically active dimeric state.

## Introduction

Aminopeptidase P (AMPP), a metalloprotease found in bacteria, yeast and mammals, specifically cleaves N-terminal amino acid residues from peptide substrates possessing proline at the neighboring (P1’) position [1–4]. Isoforms of the human enzyme include the cytosolic (hcAMPP) and membrane-bound (hmAMPP) aminopeptidase P, encoded by *XPNPEP1* and *XPNPEP2* genes, and a third hypothetical isoform thought be present in mitochondrial and cytosolic localizations [6, 7]. hcAMPP has been identified in human leukocytes and platelets, and its counterpart has been discovered in rat and guinea pig brains. hcAMPP forms a soluble homodimer of 71 kDa subunits, which is active towards long Xaa-Pro-Y peptides (such as bradykinin) and Xaa-Pro di-peptides [3, 8–11]. hmAMPP, a potential drug target for the development of cardiovascular therapeutics, shares 43% sequence identity with hcAMPP. Characterization of the hmAMPP orthologs from porcine kidney, and bovine and rat lungs has revealed that these enzymes are heavily N-glycosylated, they contain Zn^2+^, and they bind to the membrane via a glycosylphosphatidylinositol (GPI) anchor [5, 12–17]. Both cytosolic and membrane-bound AMPP isoforms are catalytically active towards bradykinin, a vasoactive peptide participating in blood pressure regulation, and selectively inhibited by the synthetic peptide apstatin [15, 18, 19].

The study reported in this manuscript focuses on the mechanism of catalysis employed by hcAMPP. The first AMPP X-ray structures, determined for the tetrameric *Escherichia coli* enzyme (*E coli* AMPP), revealed that the enzyme exists as a two-domain monomer in which the “pita-bread-fold” C-terminal domain possesses a binuclear manganese cluster [2, 20–23]. The role of the manganese cluster in catalysis is polarization of the water nucleophile, consistent with the observed preference for the Mn^2+^ in the role of cofactor, compared to Mg^2+^ or Zn^2+^ [1, 2, 4, 22]. The two metal atoms, located 3.3Å apart, are coordinated by the side chains of Asp271, Glu383, His354 and Glu406 at the “tight” binding site and the side chains of Asp260, Asp271 and Glu406 at the “loose” binding site [2, 4, 22]. The active site residues, His243, His350, and His361, perform essential roles in catalysis and in substrate binding via hydrophobic interactions with the peptide P1’ and P2’ amino acid residues [2, 22]. The more recent structure of the three-domain hcAMPP subunit (domains II and III correspond to domains I and II of the *E coli* AMPP), reported by by Li *et al*., shows that the enzyme has a conserved active site structure including the binuclear Mn^2+^ binding cleft of the catalytic domain (domain III) as well as the catalytic residues, Asp415, Asp426, Glu523, Glu537 and His489 [5]. Alignment of the hcAMPP and *E coli* AMPP structures also showed conservation of the second shell residues, His395, His485 and His498, which surround the substrate binding site [5]. Comparative analysis of the bacterial and human AMPP suggested similar mechanisms of catalysis [3, 23], including participation of a proton relay mediated by a conserved Asp-Arg-Tyr catalytic triad.

Fundamental characterization of the dynamics in proton transfer has been a key feature in mechanistic enzymology [24–28]. In the case of the AMPP proton-relay system, the side chains of residues at the active site form a hydrogen-bond network with the solvent in order to facilitate catalysis [4, 29]. Proton relay systems have been identified in a wide variety of enzymes; some examples of which are cysteine and serine proteases, NAD(P)^+^-dependent dehydrogenases, alcohol oxidases, mannitol 2-dehydrogenase from *Pseudomonas fluorescens*, endoglucanase from *Phanerochaete chrysosporium*, DNA topoisomerase 1B, and β-ketoacyl-acyl carrier protein reductase [24, 29–33]. For the purpose of investigating the mechanism of proton relay in hcAMPP catalysis, we employed a “chemical rescue”-based strategy wherein exogenous urea was used to rescue catalytic activity in the Arg mutant. Chemical rescue has proven to be is an effective tool in the systematic study of enzyme catalytic mechanisms [34]. The loss of the key functional groups of the substituted amino acids lysine, histidine, tyrosine, aspartate and arginine can be compensated by the rescue agents, amine, imidazole, phenol, acetate and guanidine, respectively [34]. For example, chemical rescue facilitated the identification of the respective roles of Asp242, Lys329 and Arg57 in catalysis by *S*. *solfataricus* α-L-fucosidase, ribulose 1,5-bisphosphate carboxylase/oxygenase and *E*. *coli* ornithine transcarbamylase [34–36].

As reported by Cottrel *et al*. in 2000, proton relay involving His429 and His532 in porcine membrane-bound AMPP (His243 and His361 are the corresponding residues in *E coli* AMPP), plays an important role in proton shuttling from the metal center to solvent during catalysis [1, 37]. More recently, we applied the chemical rescue method to provide evidence that the guanidium group of Arg404 in *E coli* AMPP forms hydrogen bonds with Asp260 and Tyr387, governing the proton transfer from solvent to the departing peptide during the cleavage of peptide bond [4]. In this study, we examined the mechanism of hcAMPP catalysis, focusing on the role played by active site residue Arg535. Based on the results obtained from chemical rescue and biophysical analysis of wild-type and mutant hcAMPP, we conclude that Arg535 functions as the key component in the proton relay system and in the stabilization of quaternary structure.

## Materials and methods

### Cloning and site mutagenesis

Recombinant human cytosolic aminopeptidase P (hcAMPP) (residues M1 to H623), having a thrombin cleavable N-terminal His-tag, was prepared by first cloning the hcAMPP encoding gene into a pET-28a vector (Novagen) using *Nde*I and *Hind* III as the restriction enzymes and the methodology described earlier [4]. To prepare the Y527F and R535A mutants, the pET28a-hcAMPP was subjected to QuikChange site mutagenesis using the primers: 5’-ATGAGCCCGGGTACTTTGAAGATGG-3’ and 5’-CCATCTTCAAAGTACCCGGGCTCAT-3’; 5’-AGATGGGGCTTTTGGAATTGCTATTGAGAATGTTGTCC-3’ and 5’-GGACAACA TTCTCAATAGCAATTCCAAAAGCCCCATCT-3’, respectively. Briefly, the PCR reaction was performed with Phusion DNA polymerase, by using a 50 μl reaction cocktail containing: 10 μl of 5X Phusion GC buffer, 1 μl of 10 mM dNTP, 1 μl of 10 μM primers, 1 μl of template, 1 μl of dimethyl sulfoxide (DMSO), 1 μl of Phusion DNA polymerase and 36 μl of ddH_2_O. Temperatures were set at 95°C, 55−70°C and 68°C for denaturation, annealing and elongation process, respectively. The mutant genes were used to transform XL1-Blue cells and were validated through DNA sequence analysis.

### Protein expression and purification

Wild-type, Y527F, and R535A hcAMPP genes, were expressed in *E*. *coli* Rosetta (DE3) competent cells. A single colony was selected from a kanamycin plate to inoculate 2 mL of LB medium containing 50 μg/mL of kanamycin, which was then incubated overnight at 37°C. The overnight culture was diluted into fresh LB medium at the ratio of 1:200 and incubated at 37°C until the culture OD600 reached 0.6. Isopropyl-β-D-thiogalactopyranoside (IPTG) was then added to a final concentration of 1 mM, followed by the 4 h induction period at 30°C. Cells were harvested by centrifugation (Hettich) at 5000 rpm and re-suspended in buffer containing10 mM Tris-HCl, 250 mM NaCl and 2.5 mM imidazole. After ultrasonication, the cell lysate was centrifugated at 5°C and 12000 rpm for 40 min. The protein was purified by affinity chromatography using a Ni^2+^ chelating column, 30 mM Tris-HCl, 30 mM imidazole wash buffer and 30 mM Tris-HCl, 300 mM imidazole elution buffer. Protein samples were concentrated and subjected to buffer exchange using 1 × PBS (pH 7.5) and an Amicon Ultra tube (Millipore) prior to the storage at −80°C. The purities of the recombinant proteins were confirmed by polyacrylamide gel electrophoresis.

### Enzyme kinetic determinations

The hydrolysis of bradykinin to des-Arg-bradykinin (Pro-Pro-Gly-Ser-Pro-Phe-Arg) catalyzed by wild-type or mutant hcAMPP, was carried at out for 15 min at 37°C using 50 mM Tris-HCl pH 7.5 as buffer, and 0.5 mM MnCl_2_ as cofactor. Reactions were terminated by heating the reaction mixture at 100°C for 10 min prior to cooling on ice. After centrifugation for 2 min, 25 μL aliquot of the supernatants were analyzed by using a reversed-phase HPLC system (Waters 2695 Separations Module) equipped with an autosampler, a UV-Vis photodiode array detector (215 nm monitoring), and a Vydac C18 column (5*μm*, 25 cm × 4.6 mm). Column elution was carried out over the course of 1 h with a gradient running from 30 to 70% (v/v) methanol in H_2_O containing 0.1% (v/v) trifluoroacetic acid. Initial velocity data were measured at varying bradykinin concentration (20–1200 μM) and then fitted using the Michaelis-Menten equation to define the steady-state kinetic constants k_cat_ and K_m_:

$$v=\frac{Vmax\;[S]}{Km+[S]}$$

where ʋ is the initial velocity, *K*_*m*_ is the Michaelis constant, *V*_*m*_ is the maximum velocity and *S* is the substrate (bradykinin) concentration. For determination of the *k*_*cat*_ and *K*_*m*_ for manganese activation, the concentration of MnCl_2_ was varied from 1–400 μM at fixed bradykinin concentration.

### Chemical rescue of hcAMPP mutants

To test the reactivation of Y527F and R535A hcAMPP kinetic assays were conducted at fixed enzyme and bradykinin concentration, and varied concentration (0–40 mM) of guanidine hydrochloride, methyl-guanidine, amino-guanidine or *N*-ethyl-guanidine in 50 mM Tris-HCl, 0.5 mM MnCl_2_, pH 7.5 at 37°C for 10 min. The percent (%) activity regained by guanidine (or guanidine derivative) was determined using the equation:

$$Chemical\;Rescue\;(\%)=\frac{\left(desBK\;\frac{\frac{uM}{min}}{ug}of\;hcAMPP\;mutants\;with\;Guanidine)-(desBK\;\frac{\frac{uM}{min}}{ug}of\;hcAMPP\;mutants\;without\;Guanidine\right)}{\left(desBK\;\frac{\frac{uM}{min}}{ug}of\;hcAMPP\;wild-type\;without\;Guanidine\right)}$$

where desBK is des-Arg-bradykinin, the hydrolytic product from bradykinin. In addition, the kinetic parameters, *K*_*m*_ and *k*_*cat*_, of R535A hcAMPP were determined at 0.1 mM, 1 mM and 10 mM of guanidine hydrochloride.

### Hydrazine effects on hcAMPP proteins

The impact of hydrazine on the activities of wild-type hcAMPP and R535A hcAMPP was determined using assay solutions initially containing 0. 5 mM bradykinin, 50 mM Tris-HCl and 0.5 mM MnCl_2_ at pH 7.5 and 37°C. Following a 10 min incubation period the enzyme, the reactions were terminated by heating the solutions to 100°C. Remaining activities were calculated using the equation denoted below:

%Remainingactivity=(amountofproductformedinthepresenceofhydrazine)/(amountofproductformedintheabsenceofhydrazine)x100

### Circular dichroism

Secondary structure analysis and thermal stability determinations were carried out for wild-type and mutant hcAMPP on hcAMPP using J-815 circular dichroism (CD) spectropolarimeter equipped with 0.1 cm path-length cuvette containing 0.1 × phosphate buffered saline (PBS). Steady state far UV-CD spectra were recorded from 250 nm to 190 nm with an average of 10 repetitive scans collected for each protein sample at the concentration of 0.35 mg/ml. Data were analyzed, averaged and converted to mean molar residue ellipticity (deg cm^2^/dmol) with the equation of [θ]_222_ = θM_MRW_/10dc, for which the mean amino acid residue weight (M_MRW_) is 112.84, the cell path in cm (d) and the concentration of protein sample in mg/ml (c). Depending on the wavelength range used, the secondary structures were analyzed using three software programs packaged in CDPro (SELCON3, CDSSTR, and CONTIN/LL) with the reference set of SP22X [38, 39]. Thermal denaturation experiments on wild-type and mutant hcAMPP protein were examined by CD using 1X PBS solvent and the temperature elevation set from 20°C to 99°C at increment of 0.5°C per interval. The enzyme conformational change was monitored by measuring the ellipticity at 222 nm. All the data were fitted with a three state model according to the equation:

$$y_{obs}=\frac{y_{N}+y_{I}\exp\left\{-\left(\Delta G_{\left(H2O\right),N-I}-m_{N-I}\left[D\right]\right)/RT\right\}+y_{U}\exp\left\{-\left(\Delta G_{\left(H2O\right),N-I}-m_{N-I}\left[D\right]\right)/RT\right\}\exp\{-(\Delta G_{\left(H2O\right),I-U}-m_{I-U}\left[D\right])/RT\}}{1+\exp\left\{-\left(\Delta G_{\left(H2O\right),N-I}-m_{N-I}\left[D\right]\right)/RT\right\}+\exp\left\{-\left(\Delta G_{\left(H2O\right),N-I}-m_{N-I}\left[D\right]\right)/RT\right\}\exp\{-(\Delta G_{\left(H2O\right),I-U}-m_{I-U}\left[D\right])/RT\}}$$

for which the native, intermediate and unfolding states are denotes as N, I and U, respectively. The observed signal (y_obs_) depends on native state signal (y_N_), intermediate state signal (y_I_), unfolding state signal (y_U_), slope (m), concentration of denaturant (D), gas constant, absolute temperature in degree kelvin and free energy (ΔG).

### Analytical ultracentrifugation

Analytical ultracentrifugation (AUC) data were collected using a XL-A analytical ultracentrifuge equipped with an An50Ti rotor (Beckman Coulter, Fullerton, CA). The 12 mm aluminum double-sector Epon centerpieces were loaded with 10 mM Tris-HCl solvent and 400 μL sample solution, respectively. The sedimentation velocity was set at 40000 rpm and the temperature at 20°C. The acquisition absorbance was set at 280 nm with the radial increment set to 0.003 cm. Time intervals of 5 min were used. Solvent density, viscosity and protein’s partial specific volumes were calculated using the software Sednterp [40]. The sedimentation coefficients and population distributions were analyzed using the Sedfit program [41].

## Results

### Purity and kinetic properties of recombinant wild-type hcAMPP and hcAMPP site-directed mutants

**Fig 1:**
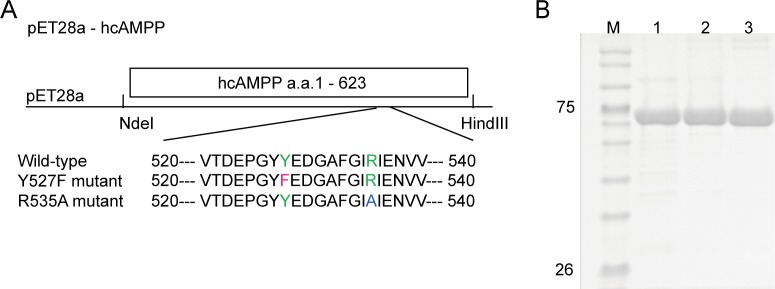
hcAMPP constructs and protein expression. **(A)** Wild-type and mutant hcAMPP orientation in the pET28a system employed in present study with the single point mutations at residue Y527 and R535, denoted as red and blue single-letter amino acid abbreviation, respectively. **(B)** hcAMPP wild-type, Y527F and R535A enzymes are shown in lane 1, 2 and 3, respectively, where M denoted the protein marker used in correspondence to show the molecular weight of each protein.

Wild-type and site-directed mutants of hcAMPP at residues 527 and 535 are illustrated in Fig 1A. DNA sequencing was employed to confirm the sequences of the Tyr527Phe and Arg535Ala hcAMPP mutant genes. The mutant genes were expressed in *E*. *coli* Rosetta (DE3) cells and the His-tagged protein products were purified using affinity chromatography. The SDS/PAGE chromatography gels (Fig 1B) confirm the protein purity and the expected approximate molecular weight of 72 kDa. All proteins were well behaved and their hydrolytic activities were unaffected by the N-terminus His-tag.

The X-ray structure of hcAMPP reported by Li *et al*. depicts a homodimer with each subunit containing an N-terminal domain, a middle domain and a C-terminal catalytic domain [5]. The hcAMPP structure revealed (*a*) the presence of two active site divalent metal ions (Mn^2+^), *(b)* dimerization mediated by the N-terminal domain and Trp477, and (*c*) conservation of the key active site residues including Asp415, Asp426, His489, Glu523, Glu537, His 395, His485 and His498 [5]. Similar to *E*. *coli* AMPP, wherein carboxylate group of Asp38 forms a hydrogen bond with the imidazole ring of His361, the Oε-1 atom of the hcAMPP Glu41 forms a hydrogen bond with the imidazole ring (at Nδ-1) of His498 [5]. In striking contrast to the *E coli* AMPPAsp38, the hcAMPP Glu41 is not required for catalysis.

In previous work we demonstrated the significant contributions made by *E*. *coli* AMPP active site residues Arg404 and Tyr387 for efficient catalysis of bradykinin hydrolysis [4]. In the present study, we measured the impact of amino acid replacement of the corresponding residues Arg535 and Tyr527 in hcAMPP. The steady-state kinetic parameters *k*_*cat*_, *K*_*m*_ *and k*_*cat*_*/K*_*m*_ determined for wild-type and mutant hcAMPP are given in Table 1. The turn over-rates (*k*_*cat*_) determined for theY527F and R535A hcAMP mutants are 6- and 12-fold lower than that of the wild-type enzyme, whereas the specificity constants (*k*_*cat*_*/K*_*m*_) are 7- and 24-fold lower. In conclusion, both residues make significant contributions to the catalytic efficiency.

**Table 1:** Steady-state kinetic parameters for wild-type and mutant hcAMPPs^a^.

Enzyme	*K*_m Bradykinin_ (μM)	*K*_m Mn_^2+^ (μM)	*k*_cat_ (s^-1^)	*k*_cat_/ *K*_m_ (M^-1^s^-1^)
Wild-Type	170 ± 20	2.2 ± 0.2	11 ± 2	6.5 × 10^4^
Y527F	200 ± 10	4 ± 1	1.8 ± 0.4	9.2 × 10^3^
R535A	350 ± 60	1.8 ± 0.1	0.9 ± 0.1	2.6 × 10^3^

^a^ Values reported are mean ± standard deviation derived from assays performed in 50 mM Tri-HCl, pH 7.5, at 37°C with the presence of bradykinin as described in experimental section.

**Fig 2:**
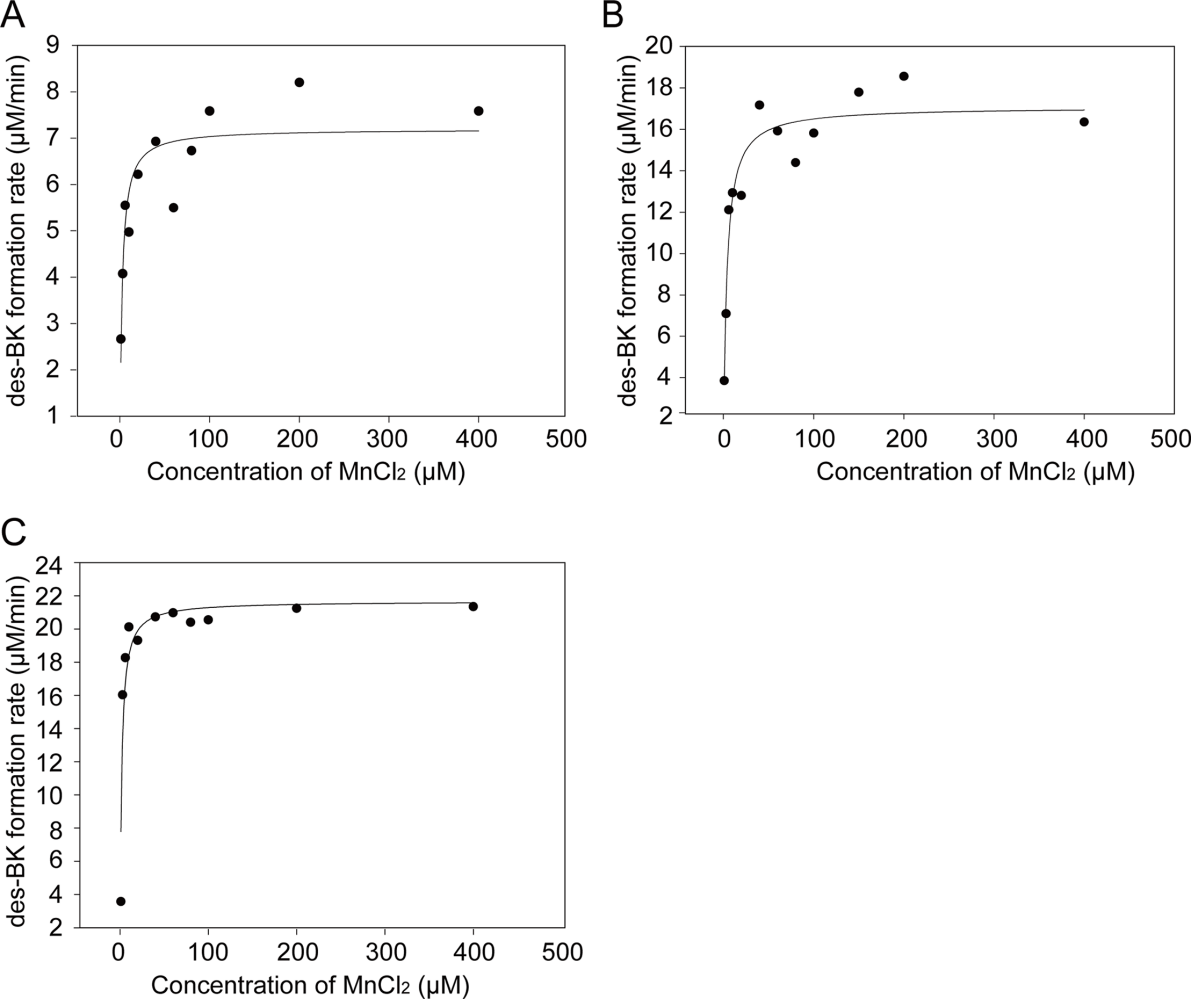
Kinetic analysis of MnCl_2_ activation of wild-type, Y527F and R535A hcAMPPs. **(A)** The rates of des-bradykinin (des-BK) product formation from bradykinin hydrolysis was plotted against the increasing concentrations of MnCl_2_ and fitted with the Michaelis-Menten equation to determine the *Km* of co-factor on hcAMPP wild-type, (**B**) Y527 mutant and (**C**) R535A mutant enzymes.

The binding affinity of the co-factor Mn^2+^ for hcAMPP was determined by measuring the *K*_*m*_ for activation, which in turn is equivalent to the Mn^2+^ dissociation constant. The initial velocity plots for Mn^2+^ activation of wild-type, Y527F and R535A hcAMPP determined at fixed bradykinin concentration are shown in Fig 2 and the Mn^2+^ *K*_*m*_ values are given in Table 1. The wild-type hcAMPP Mn^2+^ *K*_*m*_ value of 2.2 μM is essentially unchanged in the Y527F mutant (Mn^2+^ *K*_*m*_ = 1.8 μM) and increased only 2-fold in the R535A mutant to a Mn^2+^ *K*_*m*_ value of 4 μM. These results show that Y527 and R535 do not contribute significantly to cofactor binding.

### Chemical rescue of hcAMPP R535A mutant by guanidium

**Fig 3:**
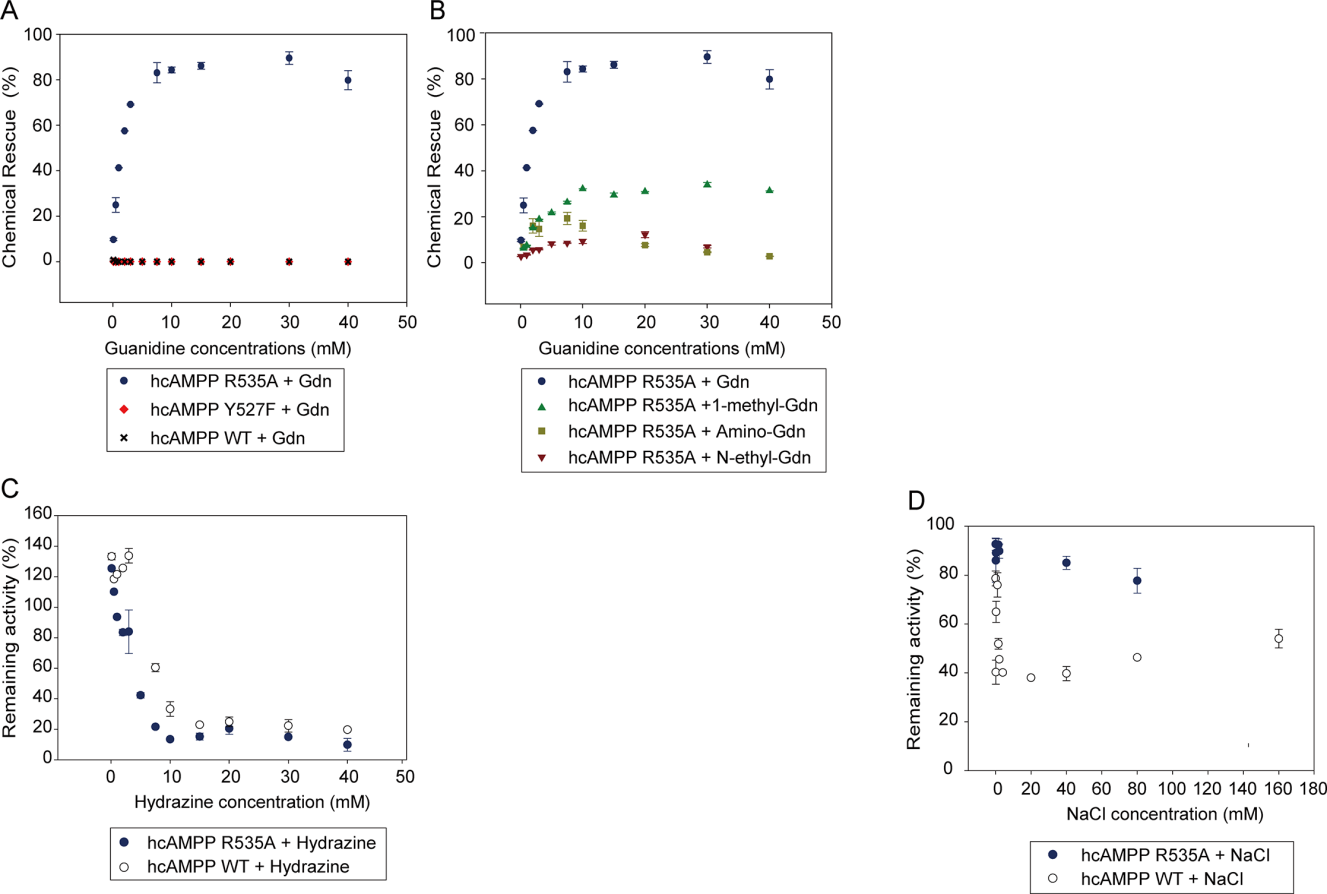
Chemical rescue of hcAMPP mutants with guanidine hydrochloride. (**A**) Guanidine hydrochloride rescue profiles for wild-type (black cross), Y527F (red diamond) and R535A (blue circle) hcAMPP. (**B**) The comparison of the profiles measured for R535A hcAMPP chemical rescue by guanidine hydrochloride (blue circle), 1-methyl guanidine (green triangle), amino-guanidine (dark-yellow rectangle) or *N*-ethyl guanidine (red inverse triangle). (**C**) The effect of varying concentrations of hydrazine on the catalytic activities of wild-type hcAMPP (open circle) and the R535A mutant (blue circle).

As reported earlier [4], the role of R404 in proton relay from the water nucleophile to solvent during *E coli* AMPP catalysis gained support from the demonstration that added guanidine significantly increased catalytic efficiency lost in the R404A mutant, whereas it had no impact on the activity of the wild-type enzyme. We applied an analogous chemical rescue technique to hcAMPP for the purpose of testing the role of Arg535 in proton relay from solvent to the peptide substrate. As expected, there was no observable rescue effect on wild-type hcAMPP nor on the Y527F mutant (Fig 3A). In contrast, approximately 90% of the activity lost in theR535A hcAMPP mutant was regained in the presence of 30 mM guanidine hydrochloride (Fig 3A and 3B). To investigate preferential interaction of different guanidine salts to R535A enzyme activation, we monitored the percent of chemical rescue at increasing concentrations using guanidine hydrochloride, methyl-guanidine, amino-guanidine or N-ethyl-guanidine. The respective levels of activity restoration were determined to be 86%, 31%, 16%, and 10% for guanidine hydrochloride, methyl-guanidine, amino-guanidine and N-ethyl-guanidine (Fig 3B). We concluded that guanidine hydrochloride is most effective and proceeded to investigate its impact on the R535A hcAMPP steady-state kinetic constants. The results reported in Table 2 reflect the dependence of the R535A hcAMPP turnover rate (*k*_*cat*_) and specificity constant (*k*_*cat*_*/K*_*m*_) on the concentration of guanidine hydrochloride. Increasing the guanidine hydrochloride concentration from 0 to 10 mM increased the *k*_*cat*_ 9-fold and the *k*_*cat*_*/K*_*m*_ 20-fold. The R535A hcAMPP *k*_*cat*_ = 8.3 s^-1^ and *k*_*cat*_*/K*_*m*_ = 5.1 x 10^4^ M^-1^ s^-1^ measured in the presence of 10 mM guanidine hydrochloride are close to those measured for wild-type where *k*_*cat*_ = 11 s^-1^ and *k*_*cat*_*/K*_*m*_ = 6.2 x 10^4^ M^-1^ s^-1^.

**Table 2:** Kinetic parameters for R535A hcAMPP measured in the presence and absence of guanidine hydrochloride^a^.

	*K*_m Bradykinin_ (μM)	*k*_cat_ (s^-1^)	*k*_cat_/ *K*_m_ (M^-1^s^-1^)
0 mM Gdn	350 ± 60	0.9 ± 0.1	2.6 × 10^3^
0.1 mM Gdn	327 ± 5	2.0 ± 0.1	6.1 × 10^3^
1 mM Gdn	144 ± 6	3.2 ± 0.1	2.2 × 10^4^
10 mM Gdn	163 ± 8	8.3 ± 0.9	5.1 × 10^4^

^a^ Values reported are mean ± standard deviation derived from assays performed in the presence of bradykinin and guanidine hydrochloride (Gdn) at 37°C in 50 mM Tri-HCl, pH 7.5, as described in experimental section.

The ability of hydrazine to rescue activity in the hcAMPP R535 mutant was also tested. The plot of the enzyme activity vs hydrazine concentration (Fig 3C) shows that hydrazine acts to inactivate wild-type hcAMPP and the R535A mutant.

### Circular dichorism (CD) spectra of purified hcAMPP mutants

**Fig 4:**
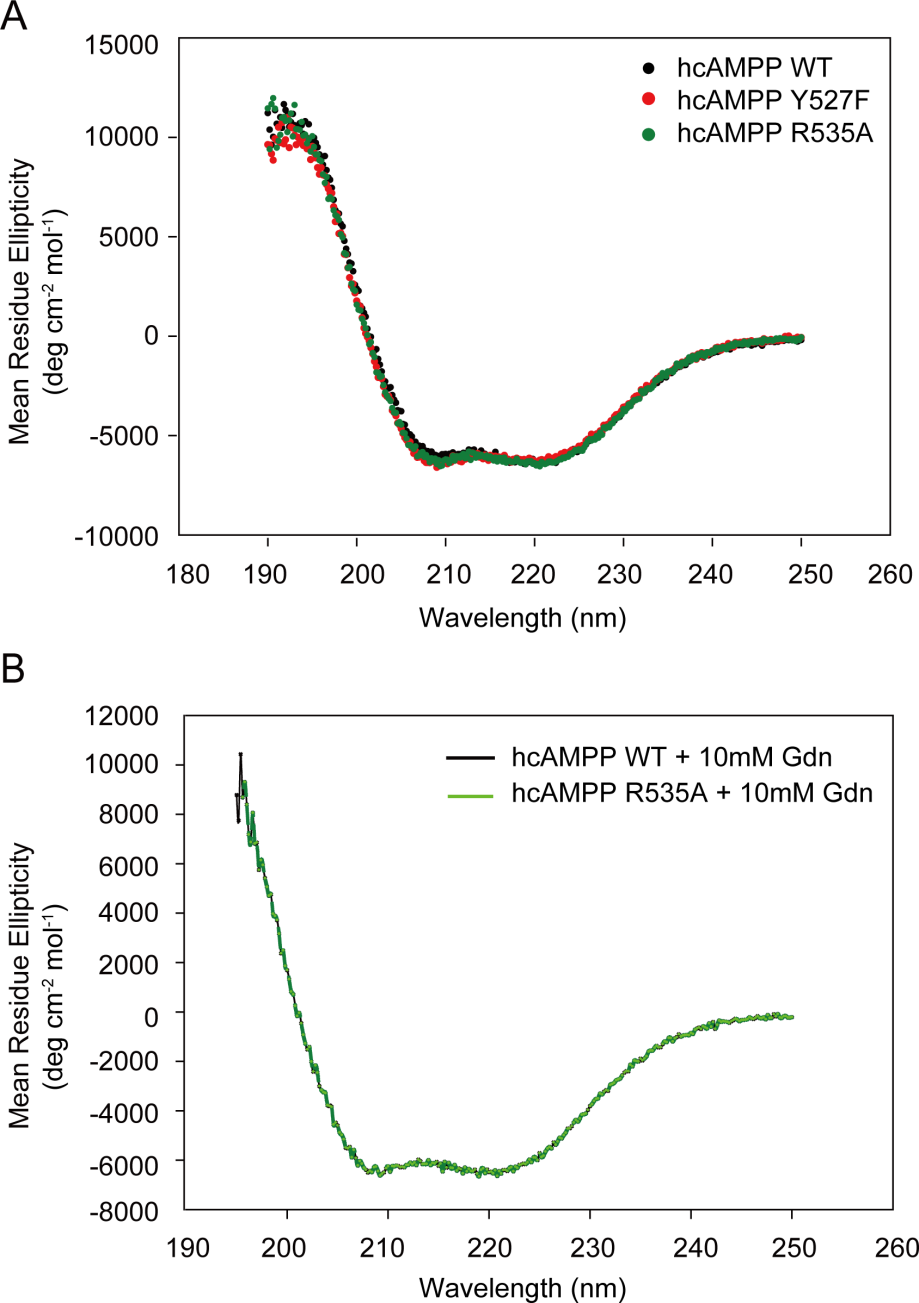
Secondary structure analyses of wild-type and mutant hcAMPPs by circular dichorism. (**A**) CD spectra of wild-type, Y527F and R535A hcAMPPs are denoted by black, red and green circles, respectively. **(B)** CD spectra of wild-type (black line) and R535A (green line) hcAMPPs measured in the presence and absence of 10 mM guanidine hydrochloride. All spectra were measured at far-UV wavelengths ranging 250–190 nm and at a protein concentration of 0.35 mg/mL.

We employed circular dichroism spectroscopy to probe changes in hcAMPP secondary structure that might result from the respective single amino acid replacements made in the wild-type hcAMPP to produce theY527F and R535A mutants. The CD spectra measured from 190 to 250 nm for the wild-type and mutant enzymes (Fig 4A) appear to be nearly identical in that all three spectra possess minima at 208 nm and 220 nm. The spectra were analyzed by using three software programs packaged in CDPro (SELCON3, CDSSTR, and CONTIN/LL) with the reference set SP22X. The assessment of wild-type hcAMPP indicated approximately 12%, 26%, 12% and 37% of α-helix, β-sheet, turns and unordered secondary structures, respectively; whereas the population of 3_10_ and polyproline helix were low, at the distribution of 6% and 5% (S1 Table). Differences between the structural propensity of hcAMPP Y527F and R535A mutants compared to that of wild-type protein were found to be insignificant (range of 0–3%). We thus concluded the hcAMPP mutants conserve the native fold of the wild-type enzyme and therefore, that the decrease in catalytic activity observed for the mutants (Table 1) can be attributed to the loss of participation by the mutated residue in catalysis rather than to a change in the backbone conformation.

Next, we used CD spectral analysis to access the impact of the presence of 10 mM guanidinium hydrochloride, which was used in the chemical rescue experiments (Table 2; Fig 3), on the secondary structure of the wild-type hcAMPP and the R535A mutant. As depicted in Fig 4A, the two CD spectra, measured from 200–250 nm, appear to be indistinguishable. The calculated secondary structure composition of the wild-type hcAMPP is consistent with the published X-ray structure [5] and in agreement with the composition calculated for the R535A hcAMPP. Specifically, in the presence of 10 mM guanidine hydrochloride both wild-type and R535A hcAMPPs are comprised of 12% α-helix and 6% polyproline helix. The percentages of β-sheet (25% for the wild-type enzyme vs 26% for the mutant enzyme) and turns (11% for the wild-type enzyme and 10% for the mutant enzyme) differ by only 1% and the percentages of 3_10_ helix and unordered secondary structure agree within 2% variation. We conclude that the 10 mM guanidinium hydrochloride used in the chemical rescue experiment did not alter the main chain conformation of the wild-type enzyme or that of the R535 mutant.

### Thermal stability of wild-type and mutant hcAMPPs

**Fig 5:**
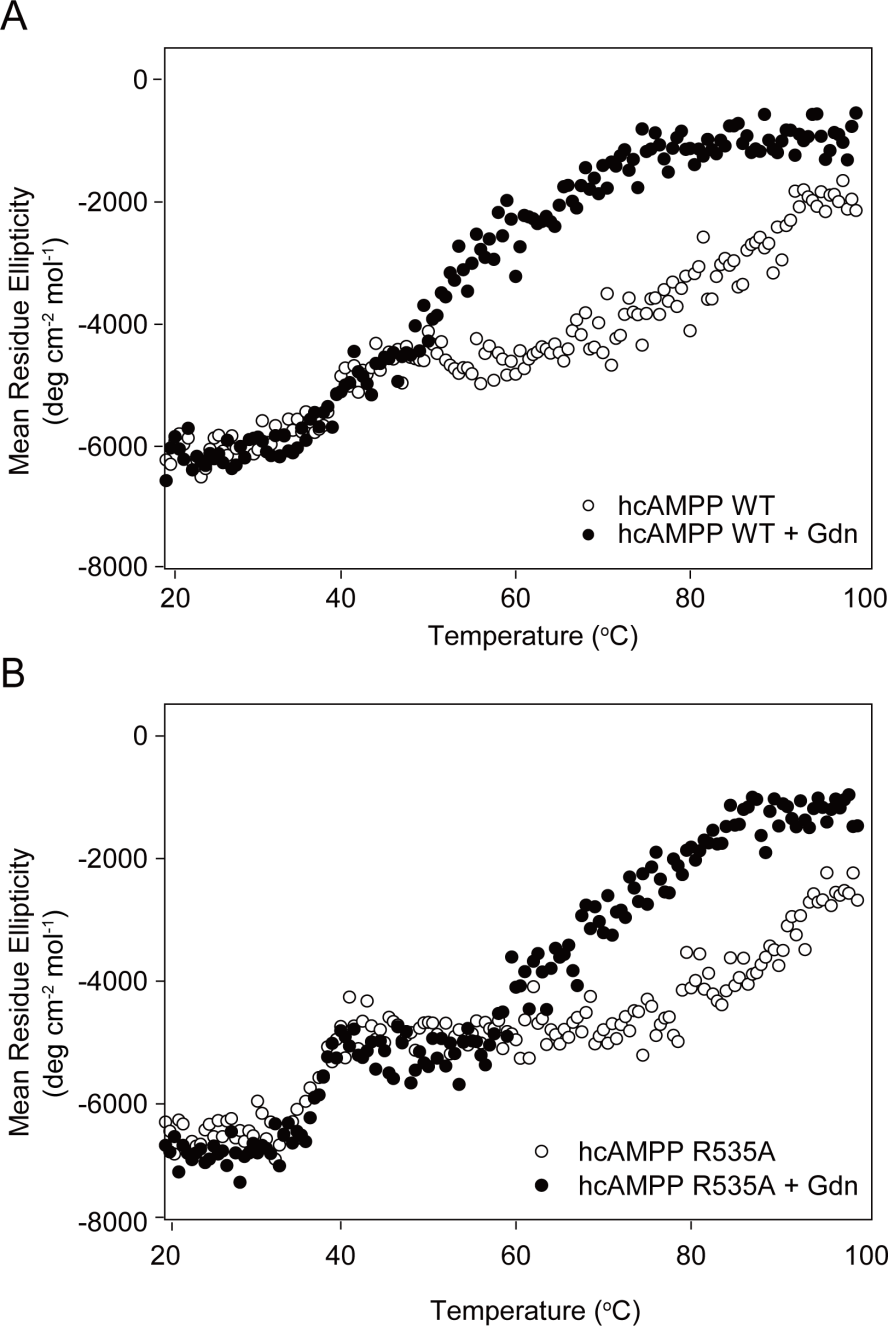
The effect of guanidine on the thermal stability of wild-type and R535A hcAMPPs monitored by circular dichorism. Temperature dependence of molar ellipticity at 222 nm measured for (**A**) wild-type hcAMPP and **(B)** R535A hcAMPP with (closed circle) or without (open circle) pre-incubation with guanidine hydrochloride.

We further examined the impact of guanidine hydrochloride on the physical properties of the wild-type and R535AhcAMPPs by measuring its impact on fold stability. For this purpose, the molar ellipticities of the respective enzymes, in the presence and absence of guanidine hydrochloride, were monitored at 220 nm as the solution temperature was incrementally increased from 20°C to 90°C. The thermal denaturation curves, depicted in Fig 5, reflect a three state-two phase thermal transition, one with an apparent T_m1_ of 39.7°C and the other with an apparent T_m2_ of 81.9°C. The addition of guanidine hydrochloride resulted in a decrease in second thermal transition point from T_m2_ = 81.9°C to a T_m2_ = 70.8°C (Table 3) for the wild-type enzyme. In contrast, the first transition was unaffected (T_m1_ = 38.4°C vs T_m1_ = 39.7°C measured in the absence of guanidine hydrochloride). The T_m1_ = 37.5°C measured for R535A hcAMPP is comparable to the T_m1_ = 39.7°C of wild-type hcAMPP, whereas the T_m2_ = 91.3°C is 10°C higher than that of the wild-type enzyme (T_m2_ = 81.9°C). In the presence of guanidine hydrochloride R535A hcAMPP T_m1_ = 39.3°C remains unchanged, however T_m2_ = 66.4°C is decreased by 24°C (compared to the 10°C decrease in T_m2_ observed for the wild-type enzyme). Based on these findings, we propose that alanine replacement of Arg535 impacts the stability of the hcAMPP native structure.

**Table 3:** Thermal stability of wild-type and R535A hcAMPPs in the presence and absence guanidine hydrochloride (Gdn)^a^.

	T_m1_			T_m2_		
	mean	stderr	n	mean	stderr	n
hcAMPP Wild-type without Gdn	39.7	1.2	2	81.9	3.0	2
hcAMPP Wild-type with Gdn	38.4	0.4	2	70.8	0.6	2
hcAMPP R535A without Gdn	37.6	0.3	2	91.3	0.4	2
hcAMPP R535A with Gdn	37.3	0.5	2	66.4	2.1	2

^a^ T_m_: melting temperature in°C. Mean and standard error and the number of replicates, n, are shown.

### Distribution of hcAMPP monomer-dimer species by sedimentation velocity

**Fig 6:**
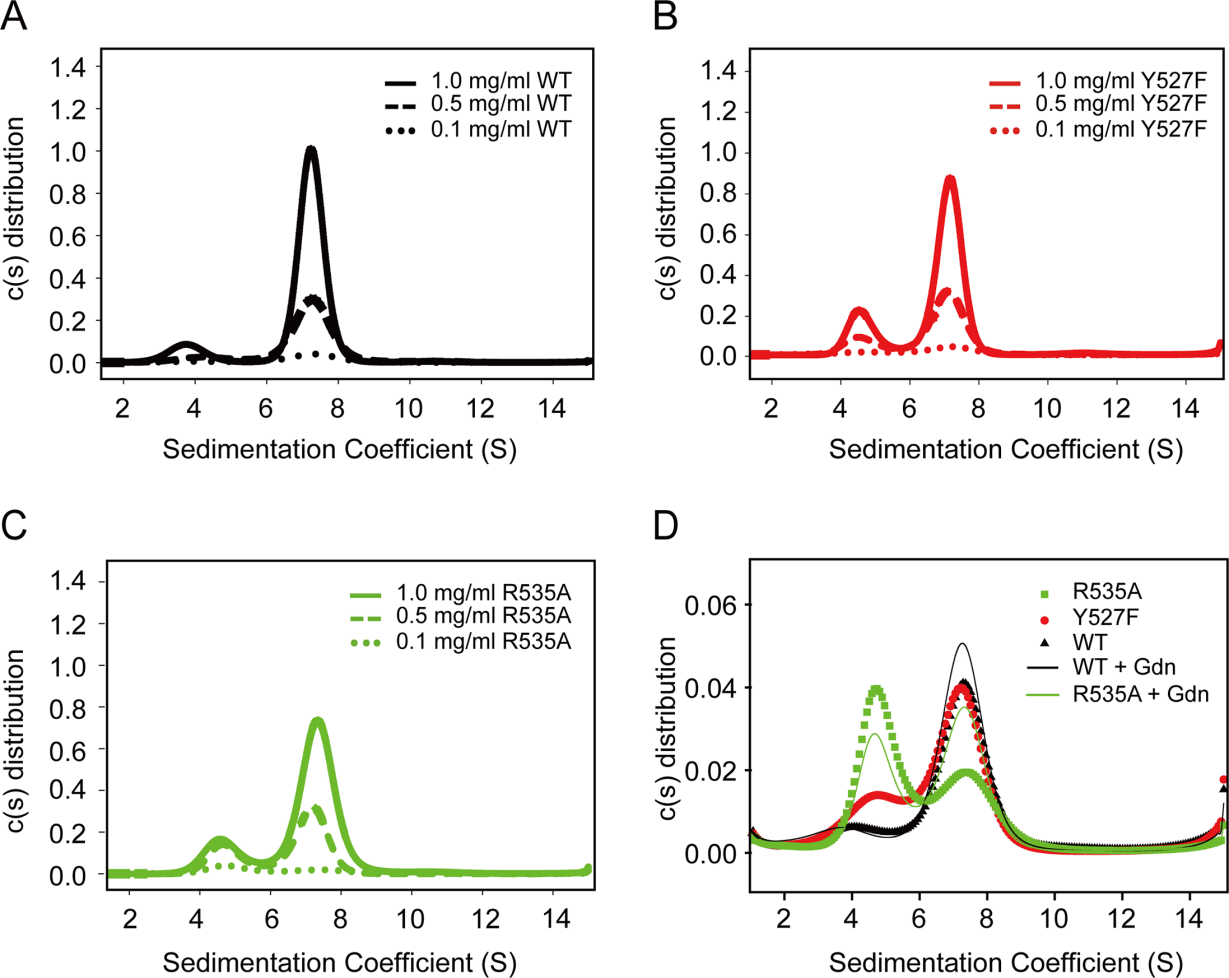
Sedimentation velocity profiles measured for wild-type and mutant hcAMPPs. The calculated hcAMPP *c(s)* distributions at the concentrations of 0.1 mg/mL (dotted line), 0.5 mg/ml (dashed line), and 1 mg/mL (solid line) for wild-type protein, Y527F mutant (red) and R535A mutant [64] are shown on (**A**), (**B**) and (**C**), respectively. (**D**) The comparison of calculated *c(s)* distributions between 0.1 mg/mL proteins of wild-type (black triangle), Y527F (red circle) and R535A (green square) hcAMPPs as well as the effect of 10 mM guanidine hydrochloride supplementation on wild-type (black line) and R535A (green line) hcAMPPs are shown.

Previous studies have revealed the importance of dimerization of hcAMPP for catalytic activity [5]. To gain insight into the prevailing quaternary structure of wild-type hcAMPP and mutant hcAMPP in solution, we employed analytical ultracentrifugation (AUC) techniques to assess the oligomerization state at different hcAMPP concentrations. The sedimentation coefficient profile measured for wild-type hcAMPP (Fig 6A) reveals the existence two species, the most abundant one being the dimer (~140 kDa), having a *S*_*20*,*w*_ = 7.48, and the other being the monomer (~ 70 kDa), having a a *S*_*20*,*w*_ = 4.53. The dimer to monomer ratio increased with wild-type hcAMPP concentration. Thus, at 0.1 mg/mL the mixture is comprised of 70% dimer and at 1 mg/mL it contains 91% of the dimer. The Y527F hcAMPP sedimentation coefficient profile shown in Fig 6B shows the dimer (*S*_*20*,*w*_ *=* 7.48) and monomer (*S*_*20*,*w*_ *=* 4.88) ratio is 3.4:1 ratio at 1 mg/mL and at a 1.8:1 measured at 0.1 mg/mL. The R535A hcAMPP sedimentation profile also contained two peaks (*S*_*20*,*w*_ values of = 7.62 and 4.98) corresponding to the dimer and monomer (Fig 6C). Alanine replacement of Arg535 in hcAMPP has a significant impact of the monomer-dimer equilibrium. At high concentration (1 mg/mL) of the mutant the dimer content is only ~70% compared to 91% for the wild-type hcAMMP, wheras at 0.1 mg/mL the monomer exceeds the dimer by 3:2. The sedimentation profiles for the wild-type, Y527F and R535A hcAMPPs measured at low concentration (0.1 mg/mL), in the presence or absence of 10 mM guanidine hydrochloride, are given in Fig 6D. The guanidine hydrochloride stabilizes the dimer relative to the monomer, an effect that is most pronounced for the R535A hcAMPP, for which the monomer:dimer ratio shifts from 1:0.67 to 1:1.5. This finding suggests that the Arg535 hydrogen bond network contributes to stabilization of the dimeric state of hcAMPP.

## Discussion

The results of previous sequence and structural comparisons have revealed that a “pita-bread” fold exist in methionine aminopeipdase, prolidase, creatinase and aminopepidase P [4, 42]. As shown in a later effort, an α-helix-wrapped β-sheet comprises the catalytic domain of *Aeromonas proteolytica* aminopeptidase, *E coli* AMPP and hcAMMP. All three aminopeptidases possess a co-catalytic di-metal center, where *A*. *proteolytica* aminopeptidase contains two Zn^2+^ ions. In contrast, *E coli* AMPP and hcAMPP contain two Mn^2+^ ion, and a co-catalytic di-Co^2+^ center is present in methionine aminopeptipdase [1, 2, 5, 22, 37, 43].

Earlier work demonstrated that in the absence of added Mn^2+^, the steady-state kinetic constants for hcAMPP-catalyzed hydrolysis of bradykinin are *K*_*m*_ = 78 μM, *k*_*cat*_ = 3.8 s^-1^ and *k*_*cat*_*/K*_*m*_ = 4.9 x 10^4^ M^-1^ s^-1^w, values that are similar to the kinetic constants (*K*_*m*_ = 101 μM, *k*_*cat*_ = 4.5 s^-1^, *k*_*cat*_*/K*_*m*_ = 4.4 x 10^4^) reported for hcAMPP catalysis occurring in solutions containing added Mn^2+^ [5, 19]. The kinetic constants determined in the present study, where assay solutions contained added Mn^2+^ are *K*_*m*_ = 170 μM, *k*_*cat*_ = 11 s^-1^, and *k*_*cat*_/*K*_*m*_ = 6.5 x 10^4^ M^-1^ s^-1^. These values are in agreement with the published values. Small differences between these data might be attributable to the difference in reaction solution pH, in view of the fact that the pH 8 is most favorable for generating the Mn^2+^-coordinated hydroxide ion nucleophile [2]. The divalent metal atoms play postulated roles in polarizing the bridging solvent molecule to form hydroxide ion that is positioned for addition to the carbonyl carbon of the Xaa-Pro peptide substrate during catalytic turnover [2, 44]. Similar to arginase (PDB:PQ3), which also requires binuclear Mn^2+^ for activity, one Mn^2+^ ion in hcAMPP has a pyramidal geometry while the other has distorted-octahedral symmetry [5, 45, 46].

As with creatinase, the catalytic domains of *E coli* AMPP and hcAMPP are linked to one or two additional domains, respectively [1, 5, 42]. Despite the difference in auxiliary domains, the catalytic domains of *E coli* AMPP and hcAMPP are structurally homologous. Although hcAMPP has a three-domain structure, it shares a conserved C-terminal catalytic domain with *E*. *coli* aminopeptidase P as demonstrated in the sequence alignment elucidated by Li et al [5]. Arginine535, in particular, is conserved in the AMPPs from humans, *E*. *coli*, *S*. *lividans*, *M*. *tuberculosis* and human or *E*. *coli* prolidases [4]. Although CD analysis demonstrated that no observable differences exist between the secondary structure compositions of wild-type hcAMPP and the R535A and Y527F mutants, the enzymatic activities of the mutants are significantly lower than that of the wild-type enzyme. We have previously shown the importance of R404 in *E*. *coli* AMPP by using guanidinium chemical rescue to facilitate hydride transfer in the hydrolysis of bradykinin. As expected, the same effect occurs in hcAMPP, but no rescue effect is observed with wild-type or Y527F mutant enzyme (Fig 3A). In contrast, the Arg535 mutant displays a maximum 90% rescue effect at 30 mM guanidine hydrochloride. Previous studies with carboxypeptidase, *E*. *coli* ornithine transcarbamylase, asparagine synthetase B, alkanesulfonate monooxygenase and phosphite dyhedrogenase have identified the participation of arginine in enzymatic catalysis by showing the existence of exogenous guanidine mediated activity restoration corresponding to an enhancement in *k*_*cat*_ [34, 47–50]. Among these, the respective R57G and R325A mutants of ornithine transcarbamylase and asparagine synthetase B also displayed preferences for rate enhancements (10% and 15%, respectively) by guanidine hydrochloride over other guanidinium based agents, which is similar to that of hcAMPP (Fig 3B) [34, 48]. As observed in the present study, increasing the concentration of guanidine hydrochloride not only raises the turn-over rate, *k*_*cat*_, but also lowers the binding affinity, *K*_*m*_ (Table 2). Therefore, activity restoration at 10 mM guanidine hydrochloride, reflected in *k*_*cat*_*/K*_*m*_, was as high as 82.3% in comparison to that of wild-type hcAMPP. Although the source of the guanidine rescue effect on the binding affinity remains unknown, the results of our circular dichorism structural analyses show that the secondary structures of wild-type and mutant enzymes remain unaffected by the presence of guanidine hydrochloride. Thus, the catalytic deficiencies caused by the R535A mutation cannot be attributed to large changes in the secondary structure content but, rather, are likely minor structural changes that are beyond the detection limit of circular dichroism detection.

The results of the pH investigation showed that R404 in *E coli* AMPP does not participate as general base in hydrolysis. Consequently, we hypothesize that R535 in hcAMPP is involved in other catalytic functions, such as hydrogen bonding [4]. This proposal was verified by the observation that low concentrations of hydrazine (H_2_NNH_2_) preserves the catalytic activity of wild-type and R535A hcAMPP. However, owing to its basic property and reducing ability, cleavage of amide bonds at high concentration of hydrazine inevitably occurs [51, 52]. We observed that the R535A mutant losses its activity to a greater extent than does the wild-type protein at the same hydrazine concentration (Fig 3C), a possible result of the lack of hydrogen bond formation caused by the missing guanidinium group in R535A. Interestingly, a similar phenomenon was observed in studies with phosphite dehydrogenase, in which the side chain of Arg301, protruding towards the active site, plays a crucial role in forming the phosphite binding entrance via water molecule mediated hydrogen bonding to Trp134 [50]. Likewise, other enzymes also utilize the arginine side chain for proper substrate binding [48, 53–57]. Hence, the interaction between the R535 guanidinium group in hcAMPP and the γ-carboxylate of Asp415, both of which are oriented towards the active site, may be the source of a suitable solvent exposed binding pocket and a 2-fold increase in *K*_*m*_ of the R535A mutant.

**Fig 7:**
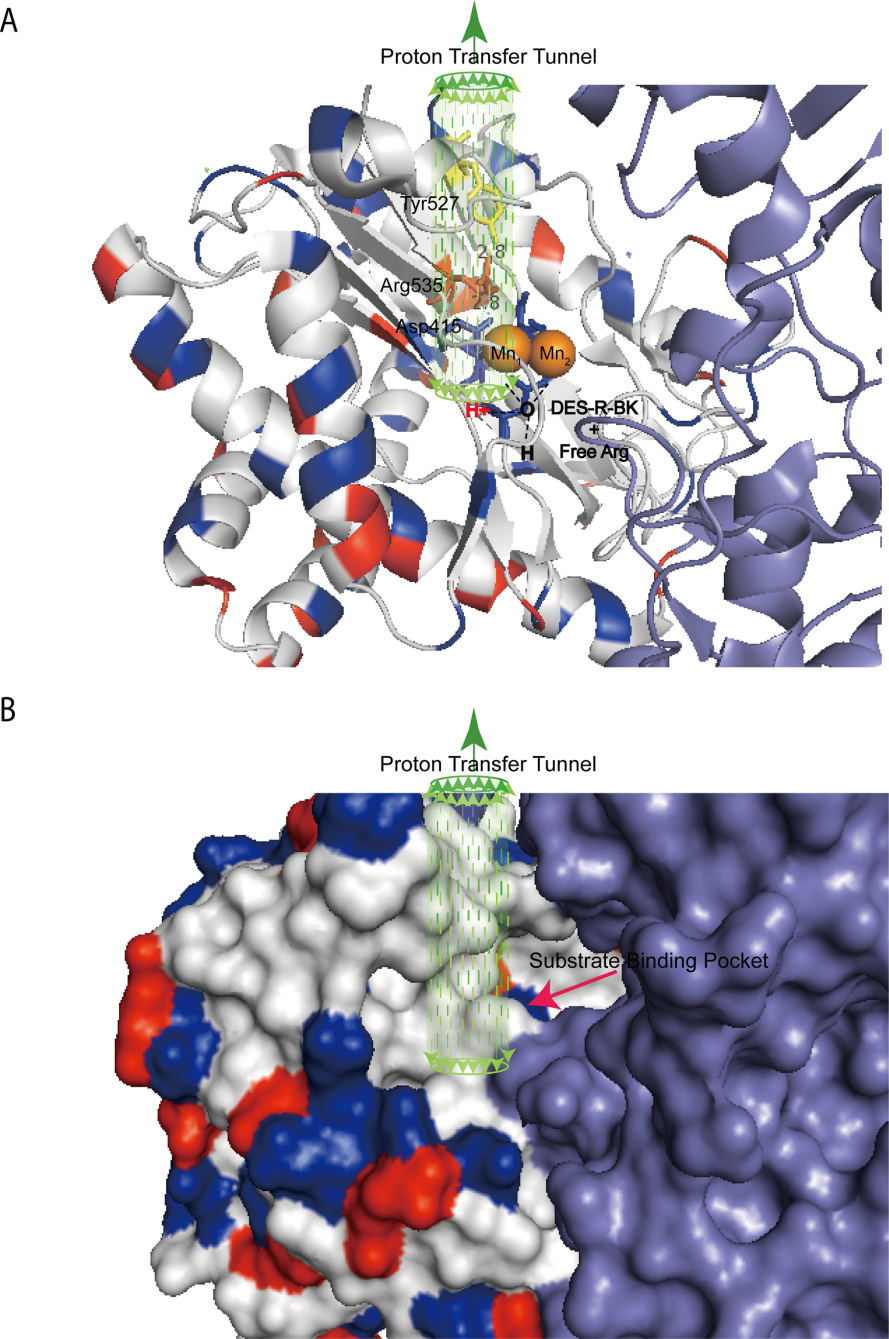
Schematic diagram of hcAMPP proton transfer tunnel using PDB code 3CTZ. Domain I and II of hcAMPP are colored in light blue whereas the catalytic domain III is colored according to hydrophobicity. Positive and negative charged residues are color coded in red and blue, respectively whereas polar and hydrophobic residues are colored in grey. **(A)** The proton transfer tunnel (green dotted arrows) is embedded in hydrophobic surrounding formed by Tyr527, Arg535 and Asp415. Proton shuttling is managed through the hydrogen bonding at 2.8Å, between the side chain of Arg535 to η-hydroxyl of Tyr527 and γ-carboxylate of Asp415. **(B)** Surface charge representation of hcAMPP shown in comparison to **(A)**.

Nonetheless, the respective 6.74-fold and 23.8-fold catalytic deficiencies detected in the Y527F and R535A mutants further demonstrate the involvement of Y527 and R535 in the proton relay system as described earlier for the *E coli* AMPP system [4]. In Fig 7A is shown a schematic diagram of the proton-relay tunnel while hydrophobicity surrounding the hydrogen transport system (PDB code 3CTZ) is portrayed in Fig 7B. In support of the proposed Mn_1_ Asp415… Arg535 … Tyr527 motif, the guanidine side chain of Arg535 is separated by 2.8Å from both side chains of the neighboring residues, forming the hydrogen bond network that facilitates proton transfer in dual directions. As observed in a variety of proton-relay systems, the guanidinium group might also play roles in transition state stabilization and proper substrate orientation for hydride transfer [48, 50, 58]. Alternatively, this network is found in essentially all X-proline-specific pita bread domain enzymes including hcAMPP. But is not present in the structurally-related methionine aminopeptidases, which are not specific for P1’ proline. It could be predicted that the roles of Arg535 and Tyr527 in this network provides a specific binding site for the substrate P1’ proline residue and orients the scissile bond for nucleophilic attack by the metal-bound hydroxide. X-ray data involving apstatin bound to cytosolic aminopeptidase P from *Caenorhabditis elegans* and *Plasmodium falciparum* support this thought [59, 60].

The above results suggest that no correlation exists between the thermal stability of hcAMPP and its activity. Compared to wild-type hcAMPP, the Arg535 mutant has a similar global structural stability, yet it has a stronger local structure corresponding to a 10 degree higher Tm_2_. Mutants having greater stabilities than wild-type proteins have been observed in previous studies [61–66]. The stability enhancement in R535A hcAMPP might be caused by the incompatibility between charged residues and the hydrophobic environment, charge-charge repulsion between R535 side chain and manganese ions and/or abolishment of interactions between R535 and the neighboring resides, Asp415 and Tyr527. Guanidine hydrochloride, which is normally used for protein denaturation through disruption of electrostatic interactions, causes a decrease in the thermal stability of the secondary structures of both wild-type and R535A proteins [47, 67–69]. The more extensive effect on the arginine mutant over that of the wild-type enzyme further suggests the crucial role played by R535 in maintaining the integrity of hcAMPP through electrostatic interactions.

hcAMPP dimer formation is mainly caused by hydrophobic interactions between tyrosine, leucine and phenylalanine residues in one subunit, and residues in the catalytic domain of the other subunit. Furthermore, salt bridges between E442 and K548 or Y549, as well as L467 and S470 contribute to dimer stabilization. We also uncovered the role played by the guanidinium group as a stabilizer for the hcAMPP dimeric quaternary structure. The R535A mutant has a diminished tendency to form a dimer but guanidine hydrochloride promotes rescue of the mutant dimer population (Fig 6D). It was hypothesized that because Y526 is involved in hydrophobic interactions between two hcAMPP subunits in the dimer, the absence of bonding between R535 and Y527 could cause a mild conformation change that indirectly affects the orientation of Y526 and Y527 that lowers the stability of the dimer. Further structural studies will be required to corroborate this proposal.

## Supporting information

S1 Table CD structure propensity. Circular dichroism analyses on the secondary structure composition of wild-type, R535A, Y527F hcAMPPs calculated from Selecton 3, Continll, CDSSTR using CDPro under the reference set of SP22X. Secondary structures predicted included α-helix, 3_10_-helix, β-sheet, turn, poly(Pro)II structure and random coil (unordered structure). Under each structural categories, average propensity (ave) and standard deviation (std) were calculated. *Gdn stands for guanidine hydrochloride in the sample.
(PDF)

## Abbreviations

hcAMPP: Human cytosolic aminopeptidase P
BK: Bradykinin
des-BK: des-Bradykinin
Tris: Trisaminomethane
PBS: Phosphate-buffered saline
CD: Circular dichroism
SDS-PAGE: Sodium dodecyl sulfate-polyacrylamide gel electrophoresis
IPTG: Isopropyl-D-thiogalactopyranoside
HPLC: High-performance liquid chromatography
PCR: Polymerase chain reaction
AUC: Analytical ultracentrifugation
