# Supplementary material for: Investigation of the proton relay system operative in human cystosolic aminopeptidase P
Source: PLoS One. 2018 Jan 19;13(1):e0190816. doi: 10.1371/journal.pone.0190816 (PMC5774706; doi:10.1371/journal.pone.0190816)
Supplement: S1 Table — Circular dichroism analyses on the secondary structure composition of wild-type, R535A, Y527F hcAMPPs calculated from Selecton 3, Continll, CDSSTR using CDPro under the reference set of SP22X. Secondary structures predicted included α-helix, 310-helix, β-sheet, turn, poly(Pro)II structure and random coil (unordered structure). Under each structural categories, average propensity (ave) and standard deviation (std) were calculated. *Gdn stands for guanidine hydrochloride in the sample. (PDF) [file pone.0190816.s001.pdf]

	α-helix	3_{10}-helix	β-sheet	Turn	Poly(Pro)II structure	Unordered
hcAMPP (Selcon3)	0.097	0.05	0.334	0.128	0.018	0.376
hcAMPP (Continll)	0.092	0.072	0.228	0.124	0.054	0.37
hcAMPP (CDSSTR)	0.157	0.064	0.223	0.114	0.074	0.361
Ave	0.12	0.06	0.26	0.12	0.05	0.37
Std	0.036	0.011	0.063	0.007	0.028	0.008
hcAMPP Y527F(Selcon3)	0.117	0.046	0.268	0.113	0.052	0.372
hcAMPP Y527F(Continll)	0.096	0.07	0.269	0.131	0.059	0.375
hcAMPP Y527F(CDSSTR)	0.152	0.057	0.225	0.102	0.082	0.366
Ave	0.12	0.06	0.25	0.12	0.06	0.37
Std	0.028	0.012	0.025	0.015	0.016	0.005
hcAMPP R535A(Selcon3)	0.068	0.037	0.341	0.109	0.039	0.393
hcAMPP R535A(Continll)	0.097	0.074	0.274	0.128	0.057	0.37
hcAMPP R535A(CDSSTR)	0.161	0.067	0.218	0.112	0.076	0.361
Ave	0.11	0.06	0.28	0.12	0.06	0.37
Std	0.048	0.020	0.062	0.010	0.019	0.017
hcAMPP_WT_Gdn (Selcon3)	0.108	0.043	0.218	0.118	0.052	0.334
hcAMPP_WT_Gdn (Continll)	0.096	0.042	0.293	0.114	0.063	0.392
hcAMPP_WT_Gdn (CDSSTR)	0.153	0.056	0.232	0.109	0.077	0.36
Ave	0.12	0.05	0.25	0.11	0.06	0.36
Std	0.030	0.008	0.040	0.005	0.013	0.029
hcAMPP_R535A_Gdn (Selcon3)	0.104	0.011	0.26	0.072	0.043	0.371
hcAMPP_R535A_Gdn (Continll)	0.091	0.038	0.296	0.12	0.06	0.396
hcAMPP_R535A_Gdn (CDSSTR)	0.153	0.052	0.238	0.107	0.074	0.368
Ave	0.12	0.03	0.26	0.10	0.06	0.38
Std	0.033	0.021	0.029	0.025	0.016	0.015

S1 Table. CD structure propensity. Circular dichroism analyses on the secondary structure propensity of wild-type, R535A, Y527F hcAMPP calculated from Selecton 3, Continll, CDSSTR using CDPro under the reference set of SP22X. Secondary structures predicted included α-helix, 3_{10}-helix, β-sheets, turns, poly(Pro)II structure and random coil (unordered structure). Under each structural categories, average propensity (ave) and standard deviation (std) were calculated. *Gdn stands for the addition of guanidine hydrochloride in the sample.
